# The Telehealth Skills, Training, and Implementation Project: An Evaluation Protocol

**DOI:** 10.2196/resprot.3613

**Published:** 2015-01-07

**Authors:** Andrew Bonney, Patricia Knight-Billington, Judy Mullan, Michelle Moscova, Stephen Barnett, Don Iverson, Daniel Saffioti, Elisabeth Eastland, Michelle Guppy, Kathryn Weston, Ian Wilson, Judith Nicky Hudson, Dimity Pond, Gerard Gill, Charlotte Hespe

**Affiliations:** ^1^Telehealth DivisionGraduate School of MedicineUniversity of WollongongWollongongAustralia; ^2^Research and Critical Analysis DivisionGraduate School of MedicineUniversity of WollongongWollongongAustralia; ^3^Exec DeanFaculty of Health, Arts and DesignSwinburne University of TechnologyHawthorn, 3122Australia; ^4^Department of Computer Science and Software DevelopmentComputing DisionUniversity of WollongongWollongongAustralia; ^5^Research DivisionInnovation Campus, Fairy MeadowUniversity of WollongongWollongongAustralia; ^6^Discipline of General PracticeSchool of Rural MedicineUniversity of New EnglandArmidaleAustralia; ^7^Public HealthGraduate School of MedicineUniversity of WollongongWollongongAustralia; ^8^Exec DeanGraduate School of MedicineUniversity of WollongongWollongongAustralia; ^9^DirectorDepartment of Rural HealthUniversity of NewcastleNewcastleAustralia; ^10^Professor of General PracticeDepartment of Rural HealthUniversity of NewcastleNewcastleAustralia; ^11^Professor of General PracticeDepartment of General PracticeDeakin UniversityWaurn PondsAustralia; ^12^Department of General Practice ResearchSchool of MedicineUniversity of Notre DameSydneyAustralia

**Keywords:** telehealth, medical education, enhanced broadband

## Abstract

**Background:**

Telehealth appears to be an ideal mechanism for assisting rural patients and doctors and medical students/registrars in accessing specialist services. Telehealth is the use of enhanced broadband technology to provide telemedicine and education over distance. It provides accessible support to rural primary care providers and medical educators. A telehealth consultation is where a patient at a general practice, with the assistance of the general practitioner or practice nurse, undertakes a consultation by videoconference with a specialist located elsewhere. Multiple benefits of telehealth consulting have been reported, particularly those relevant to rural patients and health care providers. However there is a paucity of research on the benefits of telehealth to medical education and learning.

**Objective:**

This protocol explains in depth the process that will be undertaken by a collaborative group of universities and training providers in this unique project.

**Methods:**

Training sessions in telehealth consulting will be provided for participating practices and students. The trial will then use telehealth consulting as a real-patient learning experience for students, general practitioner trainees, general practitioner preceptors, and trainees.

**Results:**

Results will be available when the trial has been completed in 2015.

**Conclusions:**

The protocol has been written to reflect the overarching premise that, by building virtual communities of practice with users of telehealth in medical education, a more sustainable and rigorous model can be developed. The Telehealth Skills Training and Implementation Project will implement and evaluate a theoretically driven model of Internet-facilitated medical education for vertically integrated, community-based learning environments

## Introduction

### Background

Ensuring an appropriately trained and resourced medical workforce for regional, rural, and remote areas is critical to enhancing the welfare of rural Australians, as it is for the welfare of rural and remote communities globally [1]. Australian medical schools frequently arrange for some (or all) of their medical students to undertake part of their training away from major metropolitan areas with the aim of producing practitioners capable of, and motivated to, practice in regional, rural, and remote locations [2]. There is evidence that this approach results in sound educational outcomes [3,4]. In addition, this approach—together with targeted recruitment of graduates from rural areas—is highly important in creating a sustainable rural medical workforce [5].

### Telehealth and Rural Medical Workforce Sustainability

Both a cause and effect of an inadequate rural medical workforce is lack of timely access to specialist advice for onsite rural health practitioners and patients [6,7]. One potential strategy for assisting rural patients and doctors who would benefit from specialist access is telehealth. Telehealth has been defined as the “use of telecommunication techniques for the purpose of providing telemedicine, medical education, and health education over a distance” [8]. For the purposes of providing support to rural primary care providers and patients, a telehealth consultation is where a patient at a general practice, with the assistance of the general practitioner (GP) or practice nurse, undertakes a consultation by videoconference with a specialist located elsewhere [9]. While telehealth consulting is reported to be underused in Australia [10], there is evidence that telehealth consulting can result in improved access to specialized health services for rural patients [11,12], upskill of rural GPs [13], improved rural workforce retention [13], significant health gains [12], and financial savings [12]. The recent Australian government-funded “Connecting Health Services with the Future” initiative was designed to address this issue by providing Medicare rebates and financial incentives for video consultations with specialists for patients outside of major metropolitan areas [9], supported by telehealth consulting standards set by the Royal Australian College of General Practitioners [14] and Australian College of Rural and Remote Medicine [15].

However, until recently, technical restrictions including requirements for necessary bespoke hardware, video cameras, and integrated services digital network (ISDN) connectivity [16] have limited most applications of telehealth in Australia, limiting access and scalability [16]. For example, most professional quality video consulting applications, up until recently, have used fully featured Web-based medical video consulting software. These applications, including Skype, provide encryption for data transmission security and Medicare Australia standards level audit trails [17]. While there is evidence that exposure to telehealth consulting improves physician attitudes to its use [18], there are many reasons for the low rates of adoption, namely insufficient training as a barrier to the uptake and use of this technology [19]. Other barriers to adoption of telehealth have been identified including lack of adequate equipment, poor connectivity, lack of telehealth consulting skills, scheduling difficulties, inadequate reimbursement schemes, and patient reluctance [20-26]. Therefore, what is required is training for health professionals to optimize the video consultation care provided [27], supported networks of health professionals using the technology [27], stakeholder involvement in implementation, and well-developed business models.

### Virtual Communities of Practice and Rural Medical Workforce Training

A further challenge to a sustainable rural workforce in Australia is securing sufficient clinical placement sites for the numbers of rural undergraduate, prevocational, and vocational trainees required by the health system [2]. A number of factors, including reduced hospital in-patient stay times, increased use of medical technology, expanding training numbers, and a recognition of the need for generalist medical exposure, have resulted in an increase in the demand for community clinical placements [28-30]. Of particular concern in Australia is the capacity of rural clinical schools to accommodate increased training numbers [2]. Thus, new paradigms of health care professional training are required as it is now not unusual for a general practice to host medical and nursing students, pre-vocational doctors in training, and vocational GP trainees.

To manage this number of learners without exhausting the clinician teachers (and while still providing quality medical care and educational training), innovative models of teaching are required. One such model is vertically integrated teaching where all levels of learner, from undergraduate to vocational, contribute to a learning environment [31]. One way of conceptualizing vertically integrated learning environments is as communities of practice (CoPs). CoPs can be defined as “groups of people who share a concern or a passion for something they do and learn how to do it better as they interact regularly” [32]. There is strong evidence from business literature that CoPs can be effective in workplace training with measureable improvements in outcomes [33]. Geographically dispersed learners may also be connected by information technology tools to form virtual communities of practice (VCoPs) [34]. In medical education, VCoPs have been shown to be perceived by GP trainees to be useful in overcoming the isolation that can accompany training in rural areas [35].

### The Role of Internet-Based Solutions for Rural Medical Workforce Challenges

Telehealth consulting has been reported to facilitate peer communication and reduce isolation for rural and remote doctors [11]. It has also been demonstrated that rurally relevant continuing medical education is effective in increasing practitioners’ confidence to practice in a rural location, reducing isolation and increasing intention to continue to work in a rural area [36]. There is also evidence that VCoPs can be effective in fostering information sharing and reducing professional isolation in GP training [37]. With the above evidence in mind, the Telehealth Skills Training and Implementation Project (TSTIP), funded through the Australian government’s Broadband Enabled Education, Skills and Services (BEES) program, aims to evaluate the introduction of two broadband Internet-based interventions in rural medical training. The first is the training of telehealth skills in medical schools coupled with the use of telehealth consulting as a teaching medium for undergraduate medical students. The second is the use of broadband-enabled “virtual clinics” to support high-quality medical education and promote vertically integrated teaching and VCoPs in rural general practice.

To guide the development of virtual communities of practice, with a view to improving knowledge sharing and overcoming medical professional isolation, the Health VCoP Framework has been developed [34]. This framework has been used in previous studies on GP training and covers the seven steps to be considered when implementing a VCoP. The steps include (1) organizing facilitation, both administrative and professional, (2) ensuring an ongoing champion and support from stakeholders, (3) establishing clear goals, (4) involving a “broad church” of users, (5) establishing a supportive environment, (6) measuring activity and progress against goals, and providing feedback to participants and opportunities for benchmarking, and (7) considering technical factors such as ease of use and access, synchronous and asynchronous interactions, and considering community factors such as providing high-quality content, building trust online, self-selection of membership, and encouragement of active and passive users.

The Health VCoP Framework will be used to help inform the project’s activities and also provide a template for assessing the success (or otherwise) of the project in fostering CoPs and VCoPs among the project’s participants. While creation of CoPs and VCoPs are aims of the project, there are other educational, technical, and administrative components of the trial that require evaluation. To aid conceptualization of the use of the Health VCoP framework for the project’s implementation and evaluation, the project plan is discussed in entirety under the Health VCoP headings in the following sections, recognizing that several measures are not relevant to the framework.

## Methods

### Project Implementation

#### Health VCoP Step 1: Organize Facilitators and Moderators—Both Clinical and Administrative

##### Virtual Clinics

To support high-quality medical education and promote vertically integrated teaching in rural general practice, we will run a series of “virtual clinics”. This will require both administrative and clinical facilitation. Clinicians associated with the University of Wollongong will provide clinical teaching, facilitating a virtual clinic that is transmitted to participating metropolitan, regional, rural and remote training sites, and to individual students and doctors who are involved in the program either through their connections with one of the universities involved or through one of the two GP training organizations involved.

The virtual clinic topics will be chosen with the aim of addressing problematic clinical issues for practice-based consulting in regional and rural areas. These sessions will be interactive with students, GP trainees, and GP preceptors in training sites being able to communicate, ask questions, discuss, and deliberate on clinical issues online in real-time. A key feature of the pedagogy of the virtual clinics will be a focus on teaching clinical reasoning in each case [38]. Pre-session reading and activities will be provided online before each session, and after session discussion will be available using interactive Web 2.0 technology. This provides an environment for creating a VCoP involving the students, GP trainees, GP preceptors, and tutors. Sessions will be recorded and made available to learners online after the live session.

A minimum of eight structured virtual clinics will be run during the trial, connected to participating GP practices, individuals, or educational facilities in rural and regional areas. Administrative facilitation will be undertaken by the TSTIP project team. Educational facilities suitable for involvement include the Shoalhaven campus of the University of Wollongong, the Southern Highlands facility of University of Wollongong School of Medicine, and the University of New England campus Armidale, New South Wales.

##### Telehealth Real-Patient Learning

Training sessions in telehealth consulting will be provided for participating practices and students. The trial will then use telehealth consulting as a real-patient learning experience for students, GP trainees, GP preceptors, and trainees. This educational model focuses on the student or GP trainee actively engaging in the patient’s journey’ [39] through the health system by being involved in the telehealth referral workup, consultation, debrief, and follow-up with consenting patients. It is also intended to provide experiential patient-based learning [40] of the utility of telehealth consulting for reducing professional isolation [11] in problematic clinical cases. It is planned that cases will then form the basis for in-practice teaching and also as subjects for the students’ and GP trainees’ reflective clinical logs. The students involved will be in the senior years of their training (third and fourth year) and are already involved in “parallel consulting” where they see patients prior to (or with) the GP preceptor, take histories, perform physical examinations, formulate management plans with the GP, and assist the GP with procedures. Patient participants will supply consent for this participation in keeping with professional guidelines [14,41]. Student involvement in telehealth consults with consenting patients will be a natural extension of their roles within the GP training practices.

#### Health VCoP Step 2: Champion and Support

The TSTIP is a multicenter phased project. The development phase began on July 1, 2012. The implementation phase, the subject of this evaluation, began on August 1, 2013 and will be finalized by April 30, 2015.

The University of Wollongong General Practice Academic Unit (GPAU) dedicated TSTIP team will provide the project champion role, engaging and ensuring ongoing support from stakeholders partners including the project consortium, which consists of University of Wollongong,

University of New England, University of Newcastle, University of Notre Dame Australia (Sydney Campus), Deakin University, Coast City Country General Practice Training, and GP Synergy.

#### Health VCoP Step 3: Establish Clear Goals

The educational goals for the TSTIP are to use broadband Internet to facilitate (1) the acquisition of medical skills, knowledge, or attitudes that improve confidence to practice in less supported medical environments [36], and (2) the development of professional networks and communities of practice to reduce the sense of professional isolation in less supported medical environments [34,35].

#### Health VCoP Step 4: A Broad Church

The primary target audience for the trial is the cohort of medical students, post-graduate pre-vocational doctors in training, GP trainees, and GPs in the regional, rural, and remote catchments of the project consortium. Collaborators in the consortium include universities with medical students in rural placements and Regional Training Providers with GP trainees in approximately overlapping rural areas, predominately in New South Wales.

#### Health VCoP Step 5: Establishing a Supportive Environment

The activities of the trial are designed to foster supportive educational interactions. The strategies employed by the trial include ensuring encouraging and positive moderation of all sponsored educational sessions, moderation of online feedback during sessions, nesting learning where possible in the GP training practices where the students and registrars are placed, and engaging the local GP supervisors and regional academic leaders in the learning processes.

#### Health VCoP Step 6: Measuring Activity and Progress and Providing Feedback

Real-time feedback to participants regarding their responses to the clinical content of the virtual clinics is part of the interactive nature and an integral aspect of the learning environment. In addition, the evaluation data from both virtual clinics and from telehealth consultations involving students and registrars that are collected as the project progresses will be used to continually review the project activities and adjust them appropriately. The evaluation approach, methodology, and metrics are discussed below.

##### Project Evaluation Approach

The experiential learning intrinsic to general practice-based clinical placements involves a mix of professional modeling and skills application, including the domains of clinical reasoning [38], evidence-based decision making, management processes, and professionalism [28,42]. The educational goals for the project’s activities reflect these learning emphases. Complex attributes, such as professionalism, are not readily assessed in closed-response type evaluations [43,44]. Hence, the normal processes for evaluating the impact of educational activities (pre- and post-intervention testing) are not readily employed in this trial. Additionally, there are many facets of the trial, for example, its impact on practices, changes in practice systems, and procedures and scalability that require evaluation in addition to the educational outcomes. In order to undertake this complex and multifaceted evaluation, the authors have chosen to incorporate the principles of a Responsive Evaluation [45]. The approach broadens the evaluation to include a wide range of stakeholder issues. The goal of a responsive evaluation is “to enhance the understanding of a program from the…perspective of insiders” [46]. Thus, there is a need to identify all stakeholders and involve them in assisting to define the criteria for the evaluation. In this evaluation, this will include representatives of the funder through to practice staff and technical experts through to specialist consultants. Evaluation will commence at the inception of the project and will run in parallel with the trial activities. Participation in the evaluation is voluntary for the project participants.

A priori criteria, developed in conjunction with the funder during the project development phase include (1) the number of learners involved, (2) educational benefit of telehealth consulting and virtual clinics, (3) the development of professional learning networks (CoPs), (4) performance of information technology network components, (5) human-technology interactions, (6) cost implications, and (7) sustainability of telehealth real-patient learning and virtual clinics.

The results of the above assessments combined with focused interview data from all stakeholders will be used to evaluate whether telehealth (clinical and educational) is a sustainable teaching and learning activity for the participating institutional organizations and GP teaching practices.

##### Methodological Approach

###### Overview

The project evaluation seeks to understand the impact of technology-mediated educational interventions in the contexts of complex social and human systems. A multisite case study offers an appropriate methodology for this purpose with the ability to provide findings with high internal validity [47,48]. A multisite case study method permits the investigation of a contemporary phenomenon within its context, where boundaries between context and phenomenon may be blurred and a variety of evidence sources are required [47,48]. This approach has the advantage of facilitating in-depth evaluation even when a limited sample size would not permit statistical generalizability of quantitative results [47]. The data to be collected from various facets of the trial in order to synthesize the case studies are discussed below.

###### Telehealth Skills Training

The telehealth consultation training sessions will be evaluated using surveys for students, GP preceptors, staff, and specialists administered at the end of the training program.

###### Activity Metrics

Data will be collected on an ongoing basis to track the learning activity for the trial. This includes numbers of participants in each component of the trial, the number of learning activities, and participant numbers. In addition, Internet usage data will be collected concerning the numbers and frequency of participants accessing and interacting with online learning activities.

###### Pre-Trial Participants’ Views, Barriers, and Facilitators

Initially, semistructured interviews will be held with stakeholders (GP preceptors, GP trainees, students, specialists, patients, and staff) in order to assist the development of evaluation parameters. Semistructured, rather than structured interviews will be used to identify a range of views, experiences, barriers, and facilitators regarding telehealth-based medical education.

###### Telehealth Consultations

After selected telehealth consultations, the participants (GP preceptor, specialist, student, and patient) will be asked to undertake a structured survey that will seek their reflections on the individual session. To the extent possible, the surveys will be conducted straight after the consultation in order to minimize recall errors [49]. In addition to data on perceptions, performance, and satisfaction, students will also be asked to reflect on the learning experience provided through the consultation. During consultations, technical system data will be collected relating to the speed of the connection, quality of images, and network/hardware/software reliability.

###### Telehealth Virtual Clinics

Similarly, data from all virtual clinic sessions will be collected from the participants (GP trainee, GP preceptor, or student) who will be asked to undertake a structured survey that will seek their reflections on the individual session. Students will also be asked to reflect on the learning experience provided through the virtual clinic session. Metrics concerning transmission speeds, numbers of users, interactions, and reliability of transmission will also be assessed. A mid-point analysis of collected data will be undertaken and adjustments to the trial introduced if required.

###### Post-Trial Participants’ Views and Evaluations

At the end of the trial period, semistructured interviews will be conducted with the GP preceptors, trainees, specialists, students, practice staff, IT support, patients, and Graduate School of Medicine (GSM) staff to obtain their overall impressions of telehealth consulting, performance of the network components, and the effectiveness of the training program. The process of inter-institution collaboration will be explored through stakeholder interviews and review of artefacts such as meeting minutes and project documents.

###### Cost Implications

Data will be collected regarding cost implications for the GSM through financial record review. The process of the inter-institution collaboration will be explored through stakeholder interviews and review of artefacts such as project documents. Outcomes for patients (health and cost-related) and doctors/practices (professional and business) will be assessed using a variety of sources including interviews and practice (not patient) records. Informal data collection will be undertaken throughout the trial concerning participants’ responses, concerns, successes, and challenges.

##### Case Study Recruitment and Data Collection

Table 1 outlines the proposed sites and estimated number of learners for the virtual clinic component of the case study. Table 2 outlines the proposed sites and estimated number of learners for the telehealth real-patient learning component of the trial. Table 3 summarizes the data to be collected and the analytical approach to the sets of data.

Descriptive analysis of quantitative data will be undertaken. Qualitative analyses will include content analysis and thematic analysis along established lines [50]. The Health VCoP Framework will be used as the theoretical framework for the qualitative analyses, as the development of communities of practice is a key hypothesized outcome of the trial [34]. All of the data sources will be synthesized and analyzed in a multicenter case study [47,48] to provide an in-depth evaluation of the trial, assess its sustainability, and guide future implementation.

**Table 1:** Proposed sites and learners for virtual clinic participation.

Site type	Locations	Number of practices/ sites	Number of learners (estimated)
GP practice	Armidale	3 practices	28
	Illawarra	8 practices	
	Shoalhaven	1 practice	
	Mudgee	1 practice	
	Grafton	1 practice	
	Southern Highlands	1 practice	
Education center	Shoalhaven; Southern Highlands University of Wollongong	2 sites	50
	Geelong (Deakin)	1 site	
	Sydney (University of Notre Dame Australia)	1 site	
	Armidale (University of New England)	1 site	

**Table 2:** Proposed number of sites and learners for the telehealth real patient learning participation in GP practice (these learners are also included in Table 1 for virtual clinics).

Locations	Number of practices/ sites	Number of learners (estimated)
Armidale	3 practices	20
Illawarra	2 practices	
Shoalhaven	2 practices	
Mudgee	2 practices	
Grafton	1 practice	
Southern Highlands	1 practice	

**Table 3:** Data collection schedule and analyses.

Data collection time point	Activity	Data collected	Anticipated number of evaluation participants	Analysis method
Baseline, at start of active trial	Telehealth consulting training sessions for participating practices and students	Anonymous pre- and post-telehealth training session surveys for GP preceptors, practice staff, and students	GP preceptors n=8	Descriptive analysis of quantitative data
			Practice staff n=10	
			Specialists n=10	
	Baseline interviews	Coded, re-identifiable, audio-recorded, and transcribed semistructured interviews with GP preceptors, GP trainees, students, specialists, patients, and staff to identify a wide range of views, experiences, barriers, and facilitators regarding telehealth-based medical education	GP preceptors n=8	Thematic analysis of interview transcripts
			GP trainees n=6	
			Students n=20	
			Specialists n=8	
			Patients n=20	
			Practice staff n=10	
During trial	Telehealth consultations as a “real-patient” learning modality	Structured, site-coded, anonymous surveys after 24 telehealth consultations over the duration of the project seeking views of the participants (GP preceptor and/or GP trainee, specialist, student) concerning the individual sessions	GP preceptors n=10	Descriptive analysis of quantitative data
			GP trainees n=6	
			Students n=20	
			Specialists n=12	
	Telehealth consultations as a “real-patient” learning modality	During the evaluated 24 telehealth consultations aggregated, anonymous technical system data will be collected relating to the speed of the connection and network/hardware/software reliability	Technical data from 24 telehealth consultations	Descriptive analysis of quantitative data
	Virtual clinics as a learning modality	Structured, site-coded, anonymous surveys after all telehealth virtual clinic sessions over the duration of the project (12 in total) seeking views of the participants (GP preceptor and/or GP trainee, specialist, student) concerning the individual sessions	GP preceptors n=10	Descriptive analysis of quantitative data
			GP trainees n=6	
			Students n=70	
			Specialists n=4	
	Virtual clinics as learning modality	During the evaluated 12 telehealth virtual clinics, anonymous, aggregated technical system data will be collected relating to the speed of the connection, network/hardware/software reliability and participant interaction	Technical data from 12 telehealth virtual clinics	Descriptive analysis of quantitative data
	All activities	Data will be collected on an ongoing basis to track the learning activity for the trial. This includes numbers of participants in each component of the trial, the number of learning activities, and participant numbers	Coded, re-identifiable data from project records	Descriptive analysis of quantitative data
	Virtual clinic online interactive activities (post-session)	Aggregated, anonymous Internet usage data will be collected concerning the numbers and frequency of participants accessing and interacting with online learning activities relating to the project (pre- and post-virtual clinic online interaction)	Anonymous data from Internet Web-site usage statistics	Descriptive analysis of quantitative data
	All activities	Coded, re-identifiable informal data collection will be undertaken throughout the trial concerning participants’ responses, concerns, successes, and challenges	Coded, re-identifiable data from researcher field notes	Thematic analysis of written field notes
Follow-up post-trial	Post-trial interviews	Coded, re-identifiable, audio-recorded, and transcribed semistructured interviews with the GP preceptors, GP trainees, specialists, students, patients, practice staff, IT support, and university staff to obtain their overall impressions of the project including the performance of the project components, the effectiveness, and acceptability of the training program and cost implications	GP preceptors n=8	Thematic analysis of interview transcripts
			GP trainees n=6	
			Students n=20	
			Specialists n=8	
			Patients n=8	
			Practice staff n=10	
			IT support n=4	
			University staff n=6	
	Post-trial evaluation	The process of the inter-institution collaboration will also be explored through review of artefacts such as project documents	Coded, re-identifiable data from project records	Thematic analysis of project artefacts
	Post-trial evaluation	Data will be collected regarding cost implications for the GSM through financial record review. Outcomes for patients (health and cost-related) and doctors/practices (professional and business) will be assessed using a variety of sources including interview data, practice records, and Medicare data	Coded, re-identifiable data from interviews and project participants’ records	Descriptive analysis of quantitative data
			Universities n=5	
			Practices n=6	Thematic analysis of interview transcripts and records
			Patients n=8	
	Post-trial evaluation	All data sources will be synthesized and analyzed in a multicenter case study approach to provide an in-depth evaluation of the trial, assess its sustainability, and guide future implementation	All data sources	Mixed-methods multisite case study analysis

#### Health VCoP Step 7: Consideration of Technical Factors

The trial has focused resources on developing user-friendly, accessible technology with an in-depth technical needs assessment of participants prior to the trial, expert information technology membership in the project executive committee, a technical subcommittee dedicated to advising on technical matters, and constant user feedback concerning the usability of the technology through session evaluation reports. The technical aspects of the project will be adjusted and refined in response to feedback and the progress of the trial. High-quality content of educational sessions will be maintained by engaging content experts across the speciality fields addressed.

#### Ethical Considerations

Ethics approval for the project has been obtained through the University of Wollongong Human Research Ethics Committee (reference HE13/238).

##### Free and Informed Consent

The purpose of the evaluation will be explained to participants by researcher or project officer at each relevant data collection point, including the fact that all responses will be coded and de-identified and that participation is completely voluntary.

##### Confidentiality of Participants’ Responses

Evaluation surveys will be anonymous. All interviews be transcribed, coded, and de-identified by a research assistant. Practice location data will not be reported, and comments reported in aggregate terms. For qualitative data, participants will be assigned a code that will link their de-identified data to a confidential and secure master sheet that will contain participants’ demographic details and location.

##### Ensuring Participants Can Withdraw From the Trial Without Detriment

All potential participants will be advised that their participation is voluntary and that they may refuse to participate or may withdraw from the study at any time without penalty.

##### Data Security

All information will be securely stored and accessible only by the project investigators.

## Results

Results will be available when the trial has been completed in 2015.

## Discussion

The TSTIP will implement and evaluate a theoretically driven model of Internet-facilitated medical education for vertically integrated, community-based learning environments. This is a pragmatic trial in working practices, with an evaluation method designed to capture the reality of outcomes, sustainability, and scalability of the project activities. Limitations of the project evaluation include a lack of data on formally assessable educational outcomes, an absence of controls, and a sample size inadequate for statistical generalizability of quantitative results. However, the evaluation should provide detailed, highly internally valid data. The results will not only inform the project’s expansion, but also be of value in informing similar initiatives elsewhere, with the goal of improving the sustainability of medical workforces and health care in rural and remote regions.

## Abbreviations

CoP: communities of practice
GP: general practitioner
GSM: Graduate School of Medicine
TSTIP: Telehealth Skills Training and Implementation Project
VCoP: virtual communities of practice
